# Comment on Giraudo et al. The Use of Cyclin-Dependent Kinase 4/6 Inhibitors in Elderly Breast Cancer Patients: What Do We Know? *Cancers* 2024, *16*, 1838

**DOI:** 10.3390/cancers16162884

**Published:** 2024-08-20

**Authors:** Steven Sorscher

**Affiliations:** 1 Oncology Division, Wake Forest Medical School, Winston-Salem, NC 27157, USA; ssorscher1@gmail.com; 2 Invitae Corp., San Francisco, CA 94103, USA; 3 BioTheranostics Inc., San Diego, CA 92121, USA

In “The Use of Cyclin-Dependent Kinase 4/6 Inhibitors in Elderly Breast Cancer Patients: What Do We Know?”, Giraudo et al. reviewed the key study results from adjuvant ribociclib (NATALEE)- and abemaciclib (monarchE)-related landmark studies [1,2,3]. The authors raised important concerns about the toxicities associated with CDK4/6 inhibitors in elderly patients.

Notably, for both NATALEE and monarchE, nearly all patients received adjuvant chemotherapy, which is generally considered to be the most feared and least proven component of adjuvant therapy in patients with early-stage, high-risk breast cancer [2,3] Although there is no clear evidence that the CDK4/6 inhibitors’ adverse effects are aggravated when preceded by chemotherapy, the review by Giraudo et al. raises the question of whether elderly patients might forgo the (neo)adjuvant chemotherapy phase of therapy. In other words, might CDK4/6 inhibitors be used instead of chemotherapy in high-risk patients without significantly compromising their outcomes?

For example, in NATALEE, patients who received ribociclib plus endocrine therapy following chemotherapy demonstrated a 5.6% absolute distant recurrence risk compared to 7.3% for those who received chemotherapy plus endocrine therapy alone (28% relative risk reduction) [2]. Furthermore, the TAILORx trial showed that patients with tumors showing Oncotype DX Recurrence Scores of 26–30 had a 51.4% lower relative distant recurrence risk with chemotherapy (5.4% versus 10.4% distant recurrence risk) [4]. Thus, the risk of recurrence might be roughly calculated as 10.4 − (10.4 × 0.28) = 7.48% if a patient chooses to receive ribociclib and forgo chemotherapy, compared to 7.48 − (0.514 × 7.48) = 3.84% if instead the patient chooses ribociclib preceded by chemotherapy, which represents only a 3.64% absolute improvement in distant disease recurrence risk from chemotherapy vs. no chemotherapy preceding ribociclib.

It is important to recognize the uncertainty involved in estimating chemotherapy’s benefit because the 3-year and 9-year follow-up results from NATALEE and TAILORx, respectively, were conflated to make the calculations [2,3]. Also, ribociclib’s benefit might depend on when the patient first received chemotherapy. Notably, for patients with tumors demonstrating lower recurrence scores on TAILORx, there was less benefit from chemotherapy for post-menopausal compared to premenopausal patients, although elderly patients in particular were not discussed [4].

Given the acute and chronic toxicities associated with chemotherapy use, the omission of chemotherapy would be favored by many patients, perhaps elderly patients in particular, if it does not offer a clinically meaningful benefit. Enrolling patients on a randomized de-escalation study will be challenging because chemotherapy has been considered a key component of the adjuvant invasive breast cancer therapy approach for patients at high recurrence risk for roughly 40 years. However, a prospective trial in which elderly patients are randomly assigned CDK4/6 inhibitors plus endocrine therapy with/without chemotherapy is needed to determine with certainty whether the absolute benefit of adjuvant chemotherapy is meaningful in elderly patients who receive CDK4/6 inhibitors.
